# Supplementary material for: A mechanistic model for long-term immunological outcomes in South African HIV-infected children and adults receiving ART
Source: eLife. 2021 Jan 14;10:e42390. doi: 10.7554/eLife.42390 (PMC7857728; doi:10.7554/eLife.42390)
Supplement: Supplementary file 1. [file elife-42390-supp1.docx]

| **Parameter name** | **Range** |
| --- | --- |
| Scaled baseline CD4 | 0-2 |
| *Scaled carrying capacities* of an HIV-infected | 0-2 |
| *Scaled carrying capacities* of healthy individuals | 0-2 |
| CD4 growth rate for HIV-infected individuals | 0-1 per µm per day |
| CD4 growth rate for healthy individuals | 0-1 per µm per day |
