# Supplementary material for: A mechanistic model for long-term immunological outcomes in South African HIV-infected children and adults receiving ART
Source: eLife. 2021 Jan 14;10:e42390. doi: 10.7554/eLife.42390 (PMC7857728; doi:10.7554/eLife.42390)
Supplement: Supplementary file 2. [file elife-42390-supp2.docx]

## Comparison of parameter estimates for adults obtained using different fitting scenarios

1. ***Individual-level parameter estimates***

**Supplementary File 1a: Individual-level parameter estimates from ratio model when healthy adults’ CD4 count is 800 cells/ul (original scenario) vs. age-dependent**

| **Parameter** | **Original mean (95% CI)** | **New mean (95% CI)** | **Relative difference between means (%)*** |
| --- | --- | --- | --- |
| ***K*** | 3.457 (3.392, 3.522) | 3.439 (3.377, 3.502) | -0.5% |
| ***Q*** | 1.125 (0.901, 1.348) | 1.167 (0.922, 1.413) | +3.7% |
| ***r*** | 3.308 (3.204, 3.412) | 3.114 (3.018, 3.211) | -5.9% |
| ***s*** | 0.0222 (0.0215, 0.0229) | 0.022 (0.021, 0.023) | 0% |
| $z_{0}$ | 0.168 (0.166, 0.171) | 0.166 (0.164, 0.169) | -1.2% |

*The percent relative difference is calculated as (new mean – original mean)/(original mean) x 100

**Supplementary File 1b: Individual-level parameter estimates from ratio model when healthy adults’ CD4 count is 800 cells/ul (original scenario) vs. 1 cell/ul**

| **Parameter** | **Original mean (95% CI)** | **New mean (95% CI)** | **Relative difference between means (%)*** |
| --- | --- | --- | --- |
| ***K*** | 3.457 (3.392, 3.522) | 3.347 (3.290, 3.403) | -3.2% |
| ***Q*** | 1.125 (0.901, 1.348) | 0.984 (0.919, 1.050) | -12.5% |
| ***r*** | 3.308 (3.204, 3.412) | 3.611 (3.489, 3.732) | +9.2% |
| ***s*** | 0.0222 (0.0215, 0.0229) | 0.023 (0.022, 0.023) | +3.6% |
| $z_{0}$ | 0.168 (0.166, 0.171) | 134.9 (133, 137) | +80197.6% |

*The percent relative difference is calculated as (new mean – original mean)/(original mean) x 100

1. ***Population-level parameter estimates***

**Supplementary File 1c: Population-level parameter estimates from ratio model when healthy adults’ CD4 count is 800 cells/ul (original scenario) vs. age-dependent**

| **Parameter** | **Original mean (95% CI)** | **New mean (95% CI)** | **Relative difference between means (%)** |
| --- | --- | --- | --- |
| ***K*** | 1.75 (1.59, 1.90) | 1.75 (1.61, 1.89) | 0% |
| ***Q*** | 0.498 (0.472, 0.525) | 0.507 (0.418, 0.596) | +1.8% |
| ***r*** | 0.49 (0.39, 0.60) | 0.506 (0.506, 0.507) | +3.3% |
| ***s*** | 0.022 (0.016, 0.028) | 0.024 (0.019, 0.028) | +9.1% |
| $z_{0}$ | 0.204 (0.183, 0.225) | 0.242 (0.241, 0.242) | +18.6% |

*The percent relative difference is calculated as (new mean – original mean)/(original mean) x 100

**Supplementary File 1d: Population-level parameter estimates from ratio model when healthy adults’ CD4 count is 800 cells/ul (original scenario) vs. 1 cell/ul**

| **Parameter** | **Original mean (95% CI)** | **New mean (95% CI)** | **Relative difference between means (%)** |
| --- | --- | --- | --- |
| ***K*** | 1.75 (1.59, 1.90) | 1.7 (1.5,1.8) | -2.9% |
| ***Q*** | 0.498 (0.472, 0.525) | 0.47 (0.39, 0.57) | -5.6% |
| ***r*** | 0.49 (0.39, 0.60) | 0.55 (0.34, 0.75) | +12.3% |
| ***s*** | 0.022 (0.016, 0.028) | 0.023 (0.018, 0.030) | +4.5% |
| $z_{0}$ | 0.204 (0.183, 0.225) | 160 (145, 178) | +78331.4% |

*The percent relative difference is calculated as (new mean – original mean)/(original mean) x 100
