# Supplementary material for: A mechanistic model for long-term immunological outcomes in South African HIV-infected children and adults receiving ART
Source: eLife. 2021 Jan 14;10:e42390. doi: 10.7554/eLife.42390 (PMC7857728; doi:10.7554/eLife.42390)
Supplement: Supplementary file 3. [file elife-42390-supp3.docx]

## Evaluation of the variance-covariance matrix for the ratio and asymptotic models.

|  |  | Diagonal var-cov matrix | | Full var-cov matrix | | Diagonal Var-cov matrix + one correlation* | |
| --- | --- | --- | --- | --- | --- | --- | --- |
| Sample | Metric | Ratio Model | Asymptotic model | Ratio Model | Asymptotic model | Ratio Model | Asymptotic model |
| Adults  (12,238) | AIC | -118,835.9 | -123,077.7 | -134,172.4 | -126,807.3 | -127,774.4 | -126,446.6 |
|  | BIC | -118,754.4 | -123,025.8 | -134,016.7 | -126,733.2 | -127,685.5 | -126,387.3 |
| Children  (1,312) | AIC | 102.4134 | -1,331.167 | **-**2,258.389 | -1,500.007 | -1,920.798 | -1,382.044 |
|  | BIC | 161.6782 | -1,293.453 | **-**2,145.247 | -1,446.13 | -1,856.146 | -1,338.943 |

** The single cross-correlation is between the asymptote and the logarithm of the rate of increase of CD4+ T-cells for the asymptotic model and between the baseline scaled CD4+ T-cells and the post-ART scaled carrying capacity, for the ratio model.*
