# Supplementary material for: A mechanistic model for long-term immunological outcomes in South African HIV-infected children and adults receiving ART
Source: eLife. 2021 Jan 14;10:e42390. doi: 10.7554/eLife.42390 (PMC7857728; doi:10.7554/eLife.42390)
Supplement: Supplementary file 4. [file elife-42390-supp4.docx]

## Table 1: Unadjusted children and adults’ estimates for the Asymptotic model, under scenarios 1 &2

|  | Children | | Adults | |
| --- | --- | --- | --- | --- |
| Model | Scenario 1 (1,312 subjects) BIC = -1523.183 | Scenario 2 (1,616 subjects) BIC = -7969.775 | Scenario 1 (12,238 subjects) BIC = -126,716.2 | Scenario 2 (14,542 subjects) BIC = -186,244.2 |
| Variable | Estimate  (CV %) | Estimate  (CV %) | Estimate  (CV %) | Estimate  (CV %) |
| *Asymptote, Asy* | 0.77  (1.3) | 0.8  (1.3) | 0.66  (0.77) | 0.58  (0.63) |
| *Intercept, R0* | 0.18  (3.6) | - | 0.14  (0.91) | - |
| *Logarithm of the rate of increase, c* | 0.12  (4.0) | 0.12  (3.9) | 0.06  (2.11) | 0.11  (2.0) |

## Table 2: Children and adults’ estimated parameters, when adjusting for sex, baseline age and baseline RNA.

|  | Children | | Adults | |
| --- | --- | --- | --- | --- |
| Model | Scenario 1 (2,204 subjects) BIC = *-3,561* | Scenario 2 (2,688 subjects) BIC = *-9,640* | Scenario 1 (12,238 subjects) BIC -133,784 | Scenario 2 (14,542 subjects) BIC -178,372 |
| Variable | Estimate  (95%CI) | Estimate  (95%CI) | Estimate  (95%CI) | Estimate  (95%CI) |
| *Scaled carrying capacity post ART* | 0.82 (0.62,1.01) | 1.14 | 3.2 (2.8, 3.5) | 2.8 (2.5, 3.1) |
| Sex*, ref is male* | - | - | 0.1 (0.11,0.19) | 0.13 (0.096,0.16) |
| Age, *month* | 0.0052 (0.004,0.006) | 0.0034 | -0.00023 (-0.0004, -0.00005) | -0.00059 (-0.0007, -0.0004) |
| log viral load | 0.089 (0.07,0.10) | 0.077 | 0.016 (0.01,0.02) | 0.034 (0.028, 0.039) |
| *Scaled carrying capacity healthy individuals* | 0.665 (0.60,0.71) | 2.4 | 0.63 (0.57,0.7) | 0.61 (0.51,0.7) |
| Sex*, ref is male* | - | - | -0.19 (-0.23, -0.15) | -0.27 (-0.34, -0.21) |
| Age, *month* | 0.002 (0.001,0.003) | -0.0019 | 0.00056 (0.0003,0.0007) | 0.00072 (0.0004,0.001) |
| *Scaled CD4+ T-cells at ART initiation* | 0.89 (0.65,1.13) | - | 0.16 (0.15, 0.18) |  |
| Sex*, ref is male* | - | - | 0.076 (0.039,0.11) |  |
| Age, *month* | -0.008 (-0.009, -0.006) | - | 0.00066 (0.0004, 0.0008) |  |
| log viral load | -0.1 (-0.12, -0.08) | - | -0.061 (-0.067, -0.056) |  |
| *Rate of growth of CD4+ T-cells in healthy individuals, cells per μl per day* | 0.043 (0.037,0.048) | 0.0026 | 0.36 (0.31, 0.4) | 0.125 (0.07,0.17) |
| Sex*, ref is male* | - | - | 0.24 (0.067,0.057) | -0.0012 (-0.0021, -0.0003) |
| Age, *month* |  | 0.0167 |  |  |
| *Rate of growth of CD4+ T-cells in ART patients, cells per μl per day* | 0.18 (0.13,0.23) | 0.16 | 0.047 (0.036,0.057) | 0.044 (0.03,0.05) |
| Sex*, ref is male* | - | - | -0.44 (-0.52, -0.37) | -0.64 (-0.71, -0.56) |
| Age, *month* | - | -0.0013 | -0.0012 (-0.0015, -0.0008) | -0.00052 (-0.0008, -0.0001) |
| log viral load | 0.059 (0.03,0.08) | 0.064 | 0.13 (0.12,0.14) | 0.19 (0.17,0.2) |
