# Supplementary material for: A mechanistic model for long-term immunological outcomes in South African HIV-infected children and adults receiving ART
Source: eLife. 2021 Jan 14;10:e42390. doi: 10.7554/eLife.42390 (PMC7857728; doi:10.7554/eLife.42390)
Supplement: Transparent reporting form [file elife-42390-transrepform.docx]

***eLife’s* transparent reporting form**

We encourage authors to provide detailed information *within their submission* to facilitate the interpretation and replication of experiments. Authors can upload supporting documentation to indicate the use of appropriate reporting guidelines for health-related research (see [EQUATOR Network](http://www.equator-network.org/%20)), life science research (see the [BioSharing Information Resource](https://biosharing.org/)), or the [ARRIVE guidelines](http://www.plosbiology.org/article/info:doi/10.1371/journal.pbio.1000412) for reporting work involving animal research. Where applicable, authors should refer to any relevant reporting standards documents in this form.

If you have any questions, please consult our Journal Policies and/or contact us: [editorial@elifesciences.org](mailto:editorial@elifesciences.org).

**Sample-size estimation**

- You should state whether an appropriate sample size was computed when the study was being designed
- You should state the statistical method of sample size computation and any required assumptions
- If no explicit power analysis was used, you should describe how you decided what sample (replicate) size (number) to use

Please outline where this information can be found within the submission (e.g., sections or figure legends), or explain why this information doesn’t apply to your submission:

The initial dataset was considerably large. Thus, the sizes of the samples used in the analysis were solely determined by the availability of the variable of interest within the initial datasets. All final samples were large enough to estimate the model parameters without any identifiability issues.

This is explained in “ART patient data” page 5, lines 103-111; and in Figure 1.

**Replicates**

- You should report how often each experiment was performed
- You should include a definition of biological versus technical replication
- The data obtained should be provided and sufficient information should be provided to indicate the number of independent biological and/or technical replicates
- If you encountered any outliers, you should describe how these were handled
- Criteria for exclusion/inclusion of data should be clearly stated
- High-throughput sequence data should be uploaded before submission, with a private link for reviewers provided (these are available from both GEO and ArrayExpress)

Please outline where this information can be found within the submission (e.g., sections or figure legends), or explain why this information doesn’t apply to your submission:

This is a modelling study, and we did not perform any experiments. The model parameters were estimated from patients’ data. The exclusion criteria for the observations used for each model are detailed in section “Baseline scaled CD4+ T-cell counts” on page 8, lines 248-268; and in Figure 1.

**Statistical reporting**

- Statistical analysis methods should be described and justified
- Raw data should be presented in figures whenever informative to do so (typically when N per group is less than 10)
- For each experiment, you should identify the statistical tests used, exact values of N, definitions of center, methods of multiple test correction, and dispersion and precision measures (e.g., mean, median, SD, SEM, confidence intervals; and, for the major substantive results, a measure of effect size (e.g., Pearson's r, Cohen's d)
- Report exact p-values wherever possible alongside the summary statistics and 95% confidence intervals. These should be reported for all key questions and not only when the p-value is less than 0.05.

Please outline where this information can be found within the submission (e.g., sections or figure legends), or explain why this information doesn’t apply to your submission:

Description of each sample included in the analysis is available in Table 1 on pages 22-23 and in the text in section “Clinical characteristics” on page 10, lines 315-333.

Statistical analysis included nonlinear mixed models. A detailed description of the models is provided under “Ratio model construction” on pages 4-5, lines 115-161. The mixed model framework is described in section “Model fitting techniques” on page 6, lines 171-198.

Estimated parameters are given in Table 3, on pages 25-26. Additional estimates are given in the Supplementary files 1 & 3.

(For large datasets, or papers with a very large number of statistical tests, you may upload a single table file with tests, Ns, etc., with reference to sections in the manuscript.)

**Group allocation**

- Indicate how samples were allocated into experimental groups (in the case of clinical studies, please specify allocation to treatment method); if randomization was used, please also state if restricted randomization was applied
- Indicate if masking was used during group allocation, data collection and/or data analysis

Please outline where this information can be found within the submission (e.g., sections or figure legends), or explain why this information doesn’t apply to your submission:

This information does not apply to this current submission as it involves a modelling study of routinely collected data. No masking was applied during the analysis as the algorithm does not requires data grouped per individual.

**Additional data files (“source data”)**

- We encourage you to upload relevant additional data files, such as numerical data that are represented as a graph in a figure, or as a summary table
- Where provided, these should be in the most useful format, and they can be uploaded as “Source data” files linked to a main figure or table
- Include model definition files including the full list of parameters used
- Include code used for data analysis (e.g., R, MatLab)
- Avoid stating that data files are “available upon request”

Please indicate the figures or tables for which source data files have been provided:

Source files are available for Appendix 1 – figures 1 and 2. These contain simulated references CD4+ T-cells values per age, for healthy children, and age and sex-dependant values for healthy adults.
